# How phenotypic matching based on neutral mating cues enables speciation in locally adapted populations

**DOI:** 10.1002/ece3.5806

**Published:** 2019-11-17

**Authors:** Richard M. Sibly, Mark Pagel, Robert N. Curnow, Jonathan Edwards

**Affiliations:** ^1^ School of Biological Sciences University of Reading Reading UK; ^2^ Department of Mathematics and Statistics University of Reading Reading UK; ^3^ Division of Medicine University College London London UK

**Keywords:** assortative mating, mate choice, parapatric speciation, phenotype matching, population genetics, sexual imprinting

## Abstract

Maynard Smith's (*American Naturalist,* 1966, 100, 637) suggestion that in some cases a prerequisite for speciation is the existence of local ecological adaptations has not received much attention to date. Here, we test the hypothesis using a model like that of Maynard Smith but differing in the way animals disperse between niches. In previous studies, males disperse randomly between niches but females stay put in their natal niche. As a first step toward generalizing the model, we here analyze the case that equal proportions of the two sexes disperse between niches before breeding. Supporting Maynard Smith's (1966) hypothesis, we find that once local adaptations are established, a neutral mating cue at an independent locus can rapidly enable speciation in populations with a suitable mechanism for phenotype matching. We find that stable ecological polymorphisms are relatively insensitive to the strength of selection, but depend crucially on the extent of dispersal between niches, with a threshold of ~5% if population sizes in two niches are equal. At higher levels of dispersal, ecological differentiation is lost. These results contrast with those of earlier studies and shed light on why parapatric speciation is limited by the extent of gene flow. Our testable model provides a candidate explanation for the rapid speciation rates, diversity of appearance and occurrence of “species flocks” observed among some African cichlids and neotropical birds and may also have implications for the occurrence of punctuational change on phylogenies.

## Graphical abstract

We show how rapid parapatric speciation can occur in populations with local ecological adaptations and a suitable mechanism for phenotype matching. The process operates through a neutral cue such as plumage coloration. Our testable model provides a candidate explanation for the rapid speciation rates, diversity of appearance, and occurrence of “species flocks” observed among some African cichlids and neotropical birds.

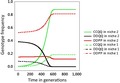

## 1 INTRODUCTION

Darwin identified the importance of mate choice in allowing females to discriminate between potential mates, but missed its potential role in avoiding cross‐species mating (Bastock, 1967). And yet, anecdotal evidence suggests the need to easily identify a same‐species mate might be commonplace. The neotropical New World oriole (*Icterus*) genus consists of around 25–30 species, similar in size, diet, and behavior but with distinct plumage coloration living in adjacent or overlapping ranges (Omland & Lanyon, 2000; Price, Friedman, & Omland, 2007). Similarly, stick insects (*Timema cristinae*) foraging on distinct co‐occurring host plants appear to be undergoing “ecological speciation” (Nosil, 2007), and the African Rift Valley lakes are home to a spectacular diversity of cichlid species, sometimes described as “species flocks” (Salzburger, Meyer, Baric, Verheyen, & Sturmbauer, 2002). Motivated by the orioles, stick insects, and cichlids, we seek here quantitative conditions for speciation, in the presence of gene flow between ecological niches, that is consistent with established mechanisms of mate choice. Two such mechanisms are sexual imprinting and phenotype matching achieved through self‐referential pleiotropy we term *Matchmaker*.

Sexual imprinting is a process by which young animals learn, generally from their parents, cues such as plumage patterns they later use to recognize potential mates. Although known in some insects, amphibians, fish, and mammals, sexual imprinting has been most studied in birds where it is thought to occur in 33 families and over half the avian orders (ten Cate & Vos, 1999). Outstanding questions in imprinting research discussed here are as follows: Why would imprinting be a widespread mechanism in mate choice (ten Cate & Vos, 1999)? And given its potential importance, who do we expect to imprint on whom (Chaffee, Griffin, & Gilman, 2013; Verzijden et al., 2012; Yang, Servedio, & Richards‐Zawacki, 2019; Yeh & Servedio, 2015)?

The other mechanism we consider, which we call *Matchmaker*, produces phenotype matching so that individuals with a novel cue (e.g., plumage coloration) prefer to mate with others with the same cue. Matchmaker involves a hypothetical self‐referent phenotype‐matching gene that affects both the expression of a cue and its recognition, in such a way that the matching system is unchanged when new mutations encode new cue patterns (Edwards & Sibly, 2017). Matchmaker is a particular candidate mechanism for mate choice in species lacking parental care, for which imprinting is unlikely. Pleiotropy in mate recognition has been demonstrated using modern genetic tools (McNiven & Moehring, 2013; Shaw, Ellison, Oh, & Wiley, 2011). For example, a single gene, *desaturase1,* plays a significant role in both emission and perception of a mating pheromone in *Drosophila melanogaster* (Bousquet et al., 2012). In *Heliconius* butterflies, a single gene encodes both female wing pattern (white or yellow spot color) and male preference for that pattern (Naisbit, Jiggins, & Mallet, 2003).

Both sexual imprinting and Matchmaker can produce assortative mating, but despite extensive theoretical effort (reviewed in Butlin et al., 2012; Gavrilets, 2004; Gavrilets, 2014; Kopp et al., 2018; Yeh & Servedio, 2015), more analysis is needed of how these mate‐choice mechanisms affect speciation in the presence of local genetic adaptation to differing ecological niches. Servedio, Saether, and Saetre (2009) analyzed the case where after allopatry hybrid matings have reduced fitness, and Yeh and Servedio (2015) analyzed learning of self‐referent phenotype matching—a proxy for some types of sexual imprinting—and showed that divergence between populations can be maintained if traits are learnt from father, but the only way the cue can spread initially is if the cue is itself under natural selection, that is, is a magic trait. Magic‐trait mate choice is known to produce speciation in the presence of ecological divergence between niches (van Doorn, Edelaar, & Weissing, 2009; Kopp et al., 2018). Here, we deal with a different case, in which the mating cue is not under natural selection.

Following a suggestion of Maynard Smith (1966), we here analyze the conditions under which assortative mating based on a neutral cue can enable speciation in a population that is already undergoing disruptive ecological selection. We follow Maynard Smith (1966) in analysing the case of two diploid loci and two niches. The two alleles at one locus confer adaptation to one or other of the two niches—we refer to this as ecological adaptation. The two alleles at the other locus control assortative mating in which individuals mate with others of the same phenotype with given probability; otherwise, they mate at random. Our model differs from Maynard Smith in the way animals disperse between niches. Maynard Smith considered the case that males disperse randomly between niches but females stay put in their natal niche and showed the possibility of speciation in a numerical simulation. Subsequent studies generalized the Maynard Smith model for cases of particular relevance to plants in which again the females do not disperse but males do (Caisse & Antonovics, 1978; Dickinson & Antonovics, 1973). Maynard Smith's model has the advantage of analytical tractability, particularly in the haploid version (Gavrilets, 2004, 2006), but it lacks generality. 40% of birds, for example, show no sex difference in at least one measure of dispersal, and overall, female birds disperse more than males (Clarke, Saether, & Roskaft, 1997). So there is need for further analysis of the effect of dispersal rules on speciation by phenotypic matching based on neutral mating cues in locally adapted populations.

As a first step to generalizing the Maynard Smith model, we here develop a diploid population genetic model and use it to analyze the case that equal proportions of the two sexes disperse between niches before breeding. Given the differences in assumptions about dispersal rules, it is not surprising we find markedly different quantitative conditions for speciation from those reported by previous workers. We consider priorities for future theoretical work, suggest field testing of predictions of where higher diversity is to be expected, and discuss the implications of our results for rates of speciation, factors promoting diversity, and the selective forces that keep the underlying neurophysiological mechanisms in place.

## 2 METHODS

**Figure 1:**
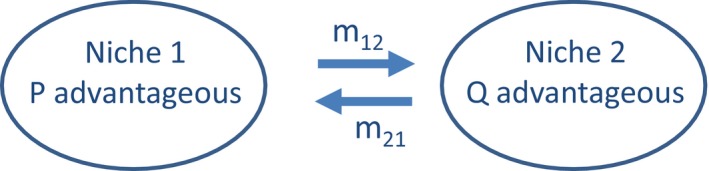
Conceptual overview of the model. For clarity, the niches are shown distinct, but in nature may be contiguous or overlap. Population sizes in each niche are constant. *m*_12_ and *m*_21_ specify the proportion of individuals in one niche that disperse to the other each generation after viability selection has occurred. Breeding occurs after dispersal. Number of offspring is determined by the product of the fitnesses of the male and the female partners. Carriers of the Q allele have fitnesses *f*_1_ and *f*_2_ in niches 1 and 2, respectively, and the fitness of PP homozygotes is set at 1 throughout

Our model has two loci and two alleles at each locus in an environment consisting of two niches with some dispersal between niches prior to mating, as depicted in Figure 1. One locus determines a mating cue, and the other locus determines ecological adaptation to one niche or the other. The two loci are unlinked and assort independently. We first describe the basic model for Matchmaker. In our model, a proportion *α* of individuals mate as directed by Matchmaker but a small proportion 1 − *α* in each niche mate at random. We assume a large population so that the dynamics are deterministic. Generations are discrete. In each generation, each individual that survives to breed produces a number of offspring proportional to the product of its fitness and that of its sole partner, and then dies. Its fitness is determined by its genotype and the niche in which it breeds.

Under Matchmaker, at the mating cue locus, allele C is dominant and, except for the random maters, its carriers mate only with other individuals carrying the C allele; the alternate allele is D. Except for random maters, DD homozygotes only mate with other DD individuals. At the unlinked ecological adaptation locus, allele Q is dominant. Its carriers obtain a fitness advantage in niche 2 (*f*_2_ > 1), but Q has lower fitness in niche 1 (*f*_1_ = 1/*f*_2_ < 1); the alternate allele is P. PP homozygotes have a fitness set to 1 in both niches.

Our method is to derive recurrence equations that give the frequencies of the nine genotypes in successive generations in each niche. To make it easier to follow, we start with the case of isolated niches and *α* = 1, and calculate the number of surviving offspring of each of the 9 × 9 = 81 possible genotype crosses in one of the niches. These are given in Table 1. The first step is to calculate the frequency of each cross, which depends on the frequencies of the two genotypes involved. For example in the top left cell, the frequency of the CCQQ × CCQQ cross is *r* ^2^ */A* (for definition of *A* see Table 1). To see this, suppose without loss of generality that females are the choosing sex. CCQQ occurs with frequency r and all CCQQ males get mates. Females carrying C only mate with males carrying C, so the proportion of the male's mates that are CCQQ is r/A, giving the frequency of the CCQQ × CCQQ cross as *r* ^2^ */A*. The number of offspring of each cross is proportional to the product of the fitnesses of its parents, so since CCQQ parents have fitness *f*, there are *f* ^2^ offspring from each CCQQ × CCQQ breeding pair. So in total in the next generation, there will be *f* ^2^ *r* ^2^ */A* individuals from the CCQQ × CCQQ cross. This is the term in the top left cell of Table 1.

**Table 1:** Relative frequencies of the genotypes of the surviving offspring of all possible genotype crosses in an isolated niche for the case *α* = 1

	CCQQ, *r*	CCQP, *s*	CCPP, *t*	CDQQ, *u*	CDQP, *v*	CDPP, *w*	DDQQ, *x*	DDQP, *y*	DDPP, *z*
CCQQ, *r*	*f* ^2^ *r* ^2^ */A*	*f* ^2^ *rs/A*	*frt/ A*	*f* ^2^ *ru/A*	*f* ^2^ *rv/A*	*frw/A*	0	0	0
CCQP, *s*	*f* ^2^ *sr/A*	*f* ^2^ *s* ^2^ */A*	*fst/A*	*f* ^2^ *su/A*	*f* ^2^ *sv/A*	*fsw/A*	0	0	0
CCPP, *t*	*ftr/A*	*fts/A*	*t* ^2^ */A*	*ftu/A*	*ftv/A*	*tw/A*	0	0	0
CDQQ, *u*	*f* ^2^ *ur/A*	*f* ^2^ * us/A*	*fut/A*	*f* ^2^ *u* ^2^ */A*	*f* ^2^ *uv/A*	*fuw/A*	0	0	0
CDQP, *v*	*f* ^2^ *vr/A*	*f* ^2^ *vs/A*	*fvt/A*	*f* ^2^ *vu/A*	*f* ^2^ *v* ^2^ */A*	*fvw/A*	0	0	0
CDPP, *w*	*fwr/A*	*fws/A*	*wt/A*	*fwu/A*	*fwv/A*	*w* ^2^ */A*	0	0	0
DDQQ, *x*	0	0	0	0	0	0	*f* ^2^ *x* ^2^ */B*	*f* ^2^ *xy/B*	*fxz/B*
DDQP, *y*	0	0	0	0	0	0	*f* ^2^ *yx/B*	*f* ^2^ *y* ^2^ */B*	*fyz/B*
DDPP, *z*	0	0	0	0	0	0	*fzx/B*	*fzy/B*	*z* ^2^ */B*

Note

The first column gives the genotypes and frequencies of fathers, and those of mothers are in the top row. The table is symmetrical. The genotypes are as follows: CCQQ, CCQP, CCPP; CDQQ, CDQP, CDPP; and DDQQ, DDQP, DDPP, and their frequencies are as follows: *r*, *s*, *t*, *u*, *v*, *w*, x, *y*, and *z*. We write *r* + *s* + *t* + *u* + *v* + *w* = *A* and *x* + *y* + *z* = *B*.

The frequencies in the next generation are obtained from Table 1. Now allowing a proportion 1 − *α* of individuals to mate at random, in an isolated niche the frequencies of offspring of each genotype at the end of the next generation, before normalization to relative frequencies, are as follows:

$$r^{\prime }=f^{2}\left(\begin{array}{c} r^{2}+sr/2+ur/2+vr/4+rs/2+s^{2}/4+us/4\\ +vs/8+ru/2+su/4+u^{2}/4+vu/8+rv/4\\ +sv/8+uv/8+v^{2}/16 \end{array}\right)/A$$

$$s^{\prime }=f\left(\begin{array}{c} fsr/2+tr+fvr/4+wr/2+frs/2+fs^{2}/2+ts/2+fus/4\\ +fvs/4+ws/4+rt+st/2+ut/2+vt/4+fsu/4+tu/2\\ +fvu/8+wu/4+frv/4+fsv/4+tv/4+fuv/8\\ +fv^{2}/8+wv/8+rw/2+sw/4+uw/4+vw/8 \end{array}\right)/A$$

$$t^{\prime }=\left(\begin{array}{c} f^{2}s^{2}/4+fts/2+f^{2}vs/8+fws/4+fst/2+t^{2}+fvt/4+\\ wt/2+f^{2}sv/8+ftv/4+f^{2}v^{2}/16+fwv/8+fsw/4\\ +tw/2+fvw/8+w^{2}/4 \end{array}\right)/A$$

$$\begin{array}{c} u^{\prime }=f^{2}\left(\begin{array}{c} ur/2+vr/4+us/4+vs/8+ru/2+su/4\\ +u^{2}/2+vu/4+rv/4+sv/8+uv/4+v^{2}/8 \end{array}\right)/A\\ \;+2\left(1-\alpha \right)\left(\begin{array}{c} f^{2}rx+1/2\;f^{2}ry+1/2\;f^{2}sx+1/4\;f^{2}sy\\ +1/2f^{2}ux+1/4f^{2}uy+1/4f^{2}vx+1/8f^{2}vy \end{array}\right) \end{array}$$

$$\begin{array}{c} v^{\prime }=f\left(\begin{array}{c} fvr/4+wr/2+fus/4+fvs/4+ws/4+ut/2\\ +vt/4+fsu/4+tu/2+fvu/4+wu/2+frv/4\\ +fsv/4+tv/4+fuv/4+fv^{2}/4+wv/4+rw/2\\ +sw/4+uw/2+vw/4 \end{array}\right)/A\\ +2\left(1-\alpha \right)\left(\begin{array}{c} 1/2f^{2}ry+frz+1/2f^{2}sx+1/2f^{2}sy+1/2fsz\\ +ftx+1/2fty+1/4f^{2}uy+1/2fuz+1/4f^{2}vx\\ +1/4f^{2}vy+1/4fvz+1/2fwx+1/4fwy \end{array}\right) \end{array}$$

$$\begin{array}{c} w^{\prime }=\left(\begin{array}{c} f^{2}vs/8+fws/4+fvt/4+wt/2+f^{2}sv/8+ftv/4\\ +f^{2}v^{2}/8+fwv/4+fsw/4+tw/2+fvw/4+w^{2}/2 \end{array}\right)/A\\ +2\left(1-\alpha \right)\left(\begin{array}{c} 1/4f^{2}sy+1/2fsz+1/2fty+tz+1/8f^{2}vy++1/4fvz\\ +1/4fwy+1/2wz \end{array}\right) \end{array}$$

$$\begin{array}{c} x^{\prime }=f^{2}\left(u^{2}/4+vu/8+uv/8+v^{2}/16\right)/A\\ +f^{2}\left(x^{2}+yx/2+xy/2+y^{2}/4\right)/B\\ +2\left(1-\alpha \right)\left(1/2f^{2}ux+1/4f^{2}uy+1/4f^{2}vx+1/8f^{2}vy\right) \end{array}$$

$$\begin{array}{c} y^{\prime }=f\left(fvu/8+wu/4+fuv/8+fv^{2}/8+wv/8+uw/4+vw/8\right)/A\\ +f\left(fyx/2+zx+fxy/2+fy^{2}/2+zy/2+xz+yz/2\right)/B\\ +2\left(1-\alpha \right)\left(\begin{array}{c} 1/4f^{2}uy+1/2fuz+1/4f^{2}vx+1/4f^{2}vy\\ +1/4fvz+1/2fwx+1/4fwy \end{array}\right) \end{array}$$

$$\begin{array}{c} z^{\prime }=\left(f^{2}v^{2}/16+fwv/8+fvw/8+w^{2}/4\right)/A\\ +\left(f^{2}y^{2}/4+fyz/2+fzy/2+z^{2}\right)/B\\ +2\left(1-\alpha \right)\left(1/8f^{2}vy+1/4fvz+1/4fwy+1/2wz\right) \end{array}$$

These equations apply to an isolated niche, but need modification if some individuals disperse between niches. We let the fitness of carriers of the dominant Q allele be *f*_1_ and *f*_2_ in niches 1 and 2, respectively, and the fitness of PP is 1 in both niches. Let the population sizes in niches 1 and 2 be *M*_1_ and *M*_2_, respectively, and let the proportion of individuals in niche 1 emigrating to niche 2 each generation after viability selection be *m*_12_, and the proportion of those in niche 2 emigrating to niche 1 be *m*_21_. If the frequency of offspring of genotype i produced in niches 1 and 2 are *N*_1i_ and *N*_2i_, respectively, then after dispersal the number in niche 1 is *M*_1_ *N*_1i_(1 − *m*_12_) + *M*_2_ *N*_2i_ *m*_21_. An analogous formula applies to niche 2, and the number after dispersal is *M*_2_ *N*_2i_(1 − *m*_21_) + *M*_1_ *N*_1i_ *m*_12_. Assuming the numbers moving in the two directions are equal, then *M*_1_ *m*_12_ = *M*_2_ *m*_21_.

**Figure 2:**
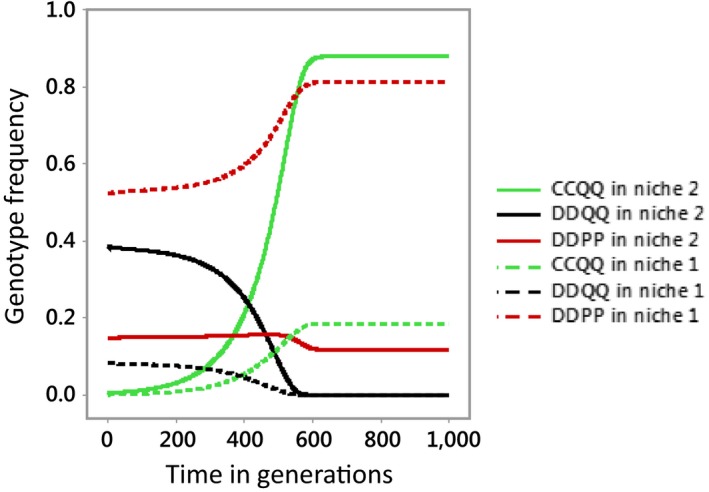
Results of running the model when the proportion of individuals in each niche that disperse to the other each generation is 3% per generation, and *α* = 1. Only three of the nine genotypes are shown. The P and Q alleles are initially at dynamic equilibrium determined by the balance between local adaptation and dispersal between the two niches as shown in Table 2. The C allele is absent before being introduced into niche 2 at a frequency of 1% CDQQ in generation 1. The fitness of carriers of Q in niche 2, *f*_2_ equals 1.1; their fitness in niche 1, *f*_1_ = 1/*f*_2_. Population sizes are the same in the two niches

To model sexual imprinting, we assume individuals imprint on the phenotype of one parent—the father, say. Suppose the original mutation is C in a male in a population of DD homozygotes. His genotype is CD, and we will suppose he mates with a DD with probability (1 − *α*) and their offspring are CD and DD, all imprinted on the C phenotype. The DD offspring from this cross are unlikely to mate (the probability is 1 − α) because they prefer a C phenotype mate but C phenotypes are unlikely to mate with them. The CDs likely mate with each other so their offspring are all imprinted on the C phenotype. These rules then continue down the generations: C phenotypes generally mate with each other, and if they have DD offspring, the DD offspring are unlikely to mate. DD genotypes generally mate with each other throughout. So there are very few mixed‐phenotype breeding pairs. The breeding scheme is like Table 1 except that DD offspring of CD × CD parents are unlikely to mate. This removes the terms divided by A in the equations for *x*′, *y*′, and *z*′ and so strengthens selection against D. This enables C to spread faster than in Figure 2.

The computer code used to run the simulations is available with explanatory annotations in Supplementary Materials. While many parameter settings are worth exploring, here we present results for dispersal rates 1, 2, 3, 4, and 5% when population sizes in the two niches are the same, and for fitnesses *f*_2_ = 1.05, 1.1 and 1.2, with in each case *f*_1_ = 1/*f*_2_ **.** Variation in population sizes is explored in Figure 4.

## 3 RESULTS

There are two alleles at the local ecological adaptation locus, P and Q, with P advantageous in niche 1 and Q in niche 2, and some dispersal between niches as shown in Figure 1. At the independent mating cue locus, there are alleles C and D, with D being fixed prior to the origination by mutation of the dominant C allele. Our model allows for a small proportion (1 − *α*) of individuals in each niche to mate at random, but we begin by presenting results for Matchmaker for the asymptotic case *α* = 1.

Before the introduction of the mating cue C allele, the population consists of genotypes DDQQ, DDPQ, and DDPP at equilibrium frequencies given by the balance between selection and dispersal (Table 2). The P allele predominates in niche 1 and the Q allele in niche 2 as a result of ecological adaptation.

**Table 2:** Equilibrium frequencies of genotypes DDQQ, DDPQ, and DDPP before the introduction of the mating cue C allele for the scenario shown in Figure 2, in which *m*_12_ = *m*_21_ = 3%, *f*_2_ = 1.1, *f*_1_ = 1/*f*_2_ and *α* = 1

	Genotype		
	DDPP	DDPQ	DDQQ
Niche 1	0.53	0.39	0.08
Niche 2	0.15	0.47	0.38

Note

**Figure 3:**
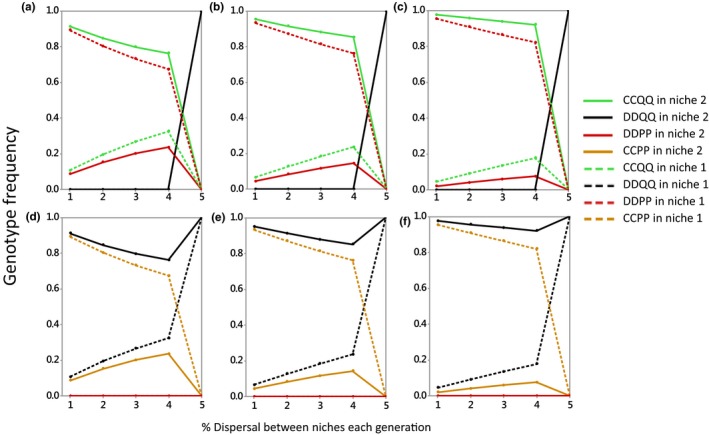
Final frequencies of key genotypes plotted against the % individuals moving between niches for three values of *f*_2_. (a and d) *f*_2_ = 1.05; (b and e) *f*_2_ = 1.1; (c and f) *f*_2_ = 1.2. In all panels, *f*_1_ = 1/*f*_2_ and *α* = 1. The P and Q alleles are initially at dynamic equilibrium, and the C allele is absent before being introduced into: top row: niche 2 at a frequency of 1% CDQQ; bottom row niche 1 at a frequency of 1% CDPP. Dots represent outputs of simulations. Population sizes are the same in the two niches

Equilibrium frequencies were calculated using our recurrence equations. PP genotypes have higher fitness than the other genotypes in niche 1 but lower fitness in niche 2; this is ecological adaptation. The asymmetry in genotype frequencies between niches result from Q being dominant to P. Results for a range of fitness effects are shown in Figure 3.

When a C allele is introduced at low frequency (1%) into niche 2 as a CDQQ genotype, it spreads rapidly (Figure 2), and the population speciates into C homozygotes and D homozygotes, with CCQQ predominating in niche 2 (solid green line), and DDPP in niche 1 (dashed red line). Eventually, all other genotypes are lost. C spreads in niche 2 because its carriers do not mate with most of the incomers from niche 1, who are predominately P‐carriers, so the population of C‐carriers has fewer disadvantageous PP genotypes than the rest of the population in niche 2.

Conversely, the C‐allele incomers migrating into niche 1 from niche 2 carry disproportionately fewer PP genotypes which alone are advantageous in niche 1, and they mate only with other C‐carrying individuals. The result is that DDPP genotypes are favoured over other genotypes in niche 1 (Figure 2, Figure 3 upper panels). The same mechanisms account for the spread of C alleles in the scenario that CDPP is introduced into niche 1 leading to spread of C alleles in niche 1 and their loss from niche 2 (Figure 3 lower panels). In both scenarios, the C allele gains an advantage over D in the niche into which it is introduced because its carriers mate less with incomers from the other niche, who carry a disproportionate load of deleterious genotypes.

Speciation here consists in replacement of the species described in Table 1, comprising genotypes DDPP, DDPQ, and DDQQ, by two species, CCQQ and DDPP, that predominate in niches 2 and 1, respectively. It is noteworthy that speciation occurs in our diploid model despite males and females migrating equally between niches, which prevents speciation in haploid models (Gavrilets, 2004, 2006).

Speciation depends upon low rates of dispersal (Figure 3). First, without dispersal, and prior to introducing mating cue C, the DDQQ genotype would have gone to fixation in niche 2 and the DDPP genotype would have done the same in niche 1. In this circumstance, if C were introduced into niche 2, it would not gain any fitness advantage over D. With moderate levels of dispersal, and for the reasons just discussed, the final outcome is two species each adapted to the niche in which it predominately lives, CCQQ in niche 2 and DDPP in niche 1 (Figure 3 upper panels). However, above ~5% dispersal speciation does not occur because ecological differentiation is lost. This illustrates how speciation driven by assortative mating depends upon the existence of polymorphism in the alleles conferring local ecological adaptation. Qualitatively, the same but opposite pattern of results occurs if the C allele is introduced at low level into niche 1 (Figure 3 lower panels). These patterns are little affected by the fitnesses of Q in the two niches (Figure 3, compare panels a, d to b, e and c, f).

**Figure 4:**
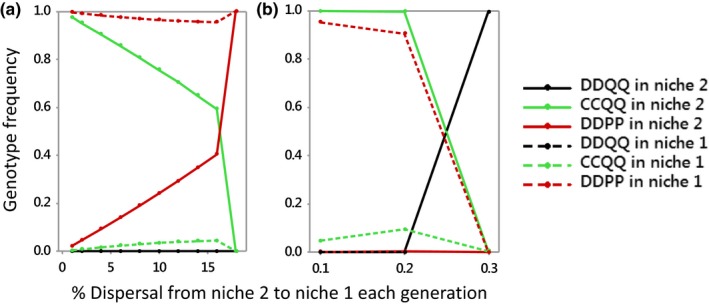
Final frequencies of key genotypes plotted against the % individuals in niche 2 that disperse to niche 1 for the case that (a) niche 1 is 10 times the size of niche 2; (b) niche 1 is one tenth the size of niche 2. Numbers moving each way are assumed the same within each panel. Symbols and initial values calculated as in Figure 3. *f*_2_ = 1.2; *f*_1_ = 1/*f*_2_ and *α* = 1. C allele absent before being introduced into niche 2 at a frequency of 1% CDQQ

Altering the relative population sizes in the two niches affects how much dispersal can occur before speciation is lost (Figure 4a,b). When the population in niche 1 is 10x larger than the population in niche 2, dispersal can be as high as 18% before speciation is lost. Conversely, if the population in niche 1 is 1/10th that of niche 2, speciation is lost at around 0.3% dispersal. The differences to the case of equal population sizes reflect the average fitnesses of DDPP genotypes and CCQQ genotypes when population sizes are unequal. With niche 1 ten times the size of niche 2, the average fitness (across niches) of P homozygotes exceeds that of Q carriers. This means that at high levels of dispersal P goes to fixation in both niches (Figure 4a), and the C allele cannot confer any advantage. Prior to that point, DDPP genotypes are sufficiently selected against in niche 2 as to allow CCQQ to predominate there. When niche 1 is 1/10th the size of niche 2, Q has higher average fitness and so at high levels of dispersal QQ goes to fixation and once again C cannot confer any mating advantage.

So far we have presented results for the asymptotic case *α* = 1 when all individuals mate as directed by Matchmaker and none mate at random. The effects of a proportion *α* < 1 in each niche mating at random are twofold: Selection of the mating cue is slower, and speciation is prevented because even when the cue reaches equilibrium, some individuals still mate at random, and this maintains all five heterozygote genotypes in the population. The effect of random mating is very small when *α* is close to one but as *α* decreases, it eventually disrupts the spread of the mating cue. The disruptive effect of random mating depends not only on *α* but also on dispersal rate. Disruption increases at higher dispersal rates because random mating then results in more heterozygous matings. For instance for *f*_2_ = 1.1, when *α* = 0.99 the final frequencies are similar to those shown in Figure 3b for dispersal rates 1%–3%, but when dispersal rate is 4%, the Q allele goes to fixation and ecological differentiation is lost.

### 3.1 Imprinting

We introduce the C allele as with Matchmaker. We assume offspring imprint on one of the parents (here, without loss of generality, fathers). We suppose that matings occur with probability *α* if the prospective mate is of the imprinted phenotype. This model differs from Matchmaker only for the case of DD offspring of CD × CD parents. These offspring, having imprinted, will seek C‐carrying mates, but the C‐carriers would not want to mate with them. As a consequence, DD offspring of CD × CD parents only mate if *α* < 1. DD offspring of DD homozygotes, however, do mate with each other. This scenario yields results numerically nearly identical to those for Matchmaker because as C spreads, CD × CD matings become less and less common.

## 4 DISCUSSION

Our results support Maynard Smith's (1966) suggestion that in some cases a prerequisite for speciation is the existence of local ecological adaptations. If a new mating cue then arises by mutation at an independent locus, given phenotype matching it can spread rapidly, producing reproductive isolation between the niches and allowing adaptive genes to spread further in the niches in which they are adaptive. Thus, the conditions for the existence of a stable ecological polymorphism dictate whether or not reproductive isolation can evolve. The conditions under which stable ecological polymorphisms can occur, in relation to dispersal rates and the strength of selection, have not received much attention in the literature. Our numerical results suggest that stable ecological polymorphisms are relatively insensitive to the strength of selection, but depend crucially on the extent of dispersal between niches, with a threshold of ~5% if the population sizes in two niches are equal (Figure 3). At higher levels of dispersal, ecological differentiation is lost and it becomes impossible for the mating cue to identify locally adapted prospective mates. More work is needed to establish, in relation to dispersal rules and cases of incomplete dominance, the conditions for stable ecological polymorphisms to occur. Analytical approaches appear to be intractable, so this may need a numerical approach.

The reason for requiring local ecological adaptation prior to the introduction of a new mating cue is that, without it, new mating cues do not increase in frequency except by genetic drift, and so speciation does not occur. With our dispersal rules, in which equal proportions of the two sexes disperse between niches before breeding, stable ecological polymorphism is only possible if dispersal rates between niches are low. By contrast in the earlier analyses of the Maynard Smith model in which males disperse at random but females stay put in their natal niches, stable ecological polymorphisms and speciation can occur whatever the level of male dispersal (Gavrilets, 2004, 2006; Maynard Smith, 1966). So dispersal rules crucially affect whether or not speciation occurs.

Our simulations show that under certain conditions mating cues spread even without complete assortative mating. Our model allows a proportion (1 − *α*) of individuals in each niche to mate not as directed by Matchmaker/Imprinting, but at random. The existence of these heterospecific matings prevents complete speciation but much of the benefit of locally adapted ecological genotypes mating with each other is still obtained if *α* is close to 1. Occasional heterospecific matings are necessary under Imprinting since otherwise an individual with a new mating cue just arisen by mutation could not find a mate. This suggests that under Imprinting there may be some optimal level of heterospecific mating, sufficient to allow individuals with new cues to find mates, but low enough to achieve near‐complete speciation and so realize the benefit of adapted ecological genotypes mating with each other. Our unreported simulations suggest the level of heterospecific matings should be less than 1%, and it would be interesting to discover from field observations its level in nature. It is perhaps relevant that the origination of a new species of Darwin's finch (Lamicchaney et al.,^2018^) in the Galapagos arose from a cross of two different species, one of which had not been seen before on the study island. After this bird bred with a bird of a resident species, their offspring mated with each other in the F1 generation, and since then, the new and the resident species have not interbred.

Sexual imprinting is widespread in nature, and our results suggest this may be because it allows mating cue alleles to enable ecological specialization. But surprisingly little is known of exactly who imprints on whom. Offspring of one or both sexes can imprint on parental phenotypes, but imprinting on siblings may also be important (Irwin & Price, 1999; ten Cate & Vos, 1999; Verzijden et al., 2012). Our approach potentially offers insight into the types of sexual imprinting that are likely to be found in nature, but further work is needed to modify the current model to compare the possibility and speed of speciation if, for example: (a) Both daughters and sons imprint on their father's phenotype, the case discussed in Results; (b) daughters imprint on their father's phenotype, sons do not imprint; (c) daughters imprint on father and sons on mother; (d) siblings imprint on siblings; and (e) imprinting on more different relatives. Preliminary analysis suggests that assortative mating is most likely if offspring imprint on one parental sex or on siblings, but is unlikely if they imprint on opposite‐sex parents or on relatives more distant than siblings. Further, theory development is needed to quantify these likelihoods and to compare our results with those of very different models (Invernizzi & Gilman, 2015; Verzijden, Lachlan, & Servedio, 2005) which suggest that speciation occurs most easily if daughters imprint on their mothers. Experiments have shown that female but not male cichlids in the genera *Pundamilia* and *Mbipia* imprint on their mothers' phenotypes (Verzijden & ten Cate, 2007; Verzijden, Korthof, & Cate, 2008; Verzijden, Zwinkels, & Cate, 2009)—as in case (b) above —raising the intriguing possibility that imprinting is part of the explanation of the spectacular speciosity of cichlids in the African rift valley lakes. Mate choice in Darwin's finches too is largely determined by sexual imprinting (Grant & Grant, 2018).

Our results might give insight into the high avian species diversity in, for example, the Neotropics, where avian species are relatively sedentary but speciose in comparison with temperate zones. If ecological variation occurs over relatively short geographical areas—affording numerous opportunities for local adaptation—natural dispersal of individuals among niches sets the stage for the evolution of mating cues and the rapid speciation we describe here. Possible examples include the high rates of speciation in the New World orioles, *Icterus*, and of endemism in the tiny Santa Marta range in north Colombia and on the eastern and western slopes of the northern Andes. In such places, parallel speciation is also theoretically possible: Genes conferring ecological adaptation to higher elevations diffuse through a population connecting isolated mountains on which different mating cues arise and produce different species on isolated mountains differing only in their mating cues and not in their ecological adaptations.

Speciation can occur at higher dispersal rates when population sizes differ (Figure 4), for example when a larger population abuts a smaller one in a different habitat, such as may have been the case in the evolution of the endemic blue chaffinch (*Fringilla teydea*) of Tenerife. This species currently lives in a relatively small area of pine forest at high elevations where its deeper beak gives it an advantage cracking pine nuts compared to the chaffinch *Fringilla coelebs* with whom it shares a common ancestor (Grant, 1979).

Sexual imprinting and/or Matchmaker pleiotropy allow speciation to occur at times when opportunities are afforded by local ecological adaptation. We therefore expect over evolutionary time to see patterns of rapid parapatric speciation in between longer stable periods—a form of punctuational evolution. Such punctuational changes have been detected in phylogenetic analyses (e.g., McEntee, Tobias, Sheard, & Burleigh, 2018; Pagel, Venditti, & Meade, 2006), and in birds Mason et al. (2017) have shown that evolutionary bursts in rates of speciation coincide with bursts in song evolution in two clades of co‐distributed passerines. When selection acts on new cues, the genes coding for the underlying neurophysiology are selected too, helping to maintain the mate‐choice mechanisms intact. Further work is needed to analyze this phenomenon, perhaps deploying the methods of Chaffee et al. (2013) to incorporate costs of being choosy, which will make speciation harder (Gavrilets, 2014).

The novel insights above suggest several quantitative predictions which require testing. Tests should include the following: (a) Analysis of the bird literature to estimate dispersal distances, from natal to breeding locations, in relation to species' ranges. This analysis is needed because theoretically speciation depends on dispersal between niches being low as shown in Figures 3 and 4. Our idea is that species with wider ranges are likely on average to contain more ecological differentiation and so would have speciated unless they had larger dispersal distances. So wide range should go with further dispersal. Preliminary results show some support (Claramunt, Derryberry, Remsen, & Brumfield, 2012; Weeks & Claramunt, 2014). (b) Phylogenetic analyses of species plumage colors to test the predictions that (i) some color traits that distinguish species evolved within the range of the ancestral species. This is worth investigating because the new theory suggests that speciation can occur within species that contain local ecological adaptations. (ii) Color traits evolved at the time of speciation. This is predicted because given local ecological adaptations, it is the evolution of the novel color traits that drives speciation.

Our analysis of a simple population genetic model has wide implications for the study of biodiversity hotspots which may occur where dispersal distances are short, for rates of speciation, and for the study of mate choice, which may play an important evolutionary role and contribute to the generation of biodiversity (Kozak, Head, & Boughman, 2011; Verzijden et al., 2012). It is possible that we might even be able to witness incipient speciation related to mate choice in populations such as birds where heritable song dialects demarcate adjacent populations (Slabbekoorn & Smith, 2002).

## CONFLICT OF INTEREST

The authors declare no competing financial interests.

## AUTHORS' CONTRIBUTIONS

This paper developed from an original idea by JE. All authors contributed to development of the model, RMS ran the simulations, and all authors contributed to writing the paper.

## Data Availability

There are no data associated with this paper.
