# Correction to “How phenotypic matching based on neutral mating cues enables speciation in locally adapted populations”

**DOI:** 10.1002/ece3.10497

**Published:** 2023-09-05

Sibly, R. M., Pagel, M., Curnow, R. N., Edwards, J., 2019. How phenotypic matching based on neutral mating cues enables speciation in locally adapted populations. Ecology and Evolution 9, 13506–13514, doi:10.1002/ece3.5806.

In paragraph 4 of the Methods section, the equations, for rʹ, sʹ, …, zʹ, contain errors for the case α < 1. These errors do not affect the reported results and are corrected in Sibly, R. M., Curnow, R. N., 2022. Sexual imprinting leads to speciation in locally adapted populations. Ecology and Evolution 12, doi:10.1002/ece3.9479.

We apologize for this error.

